# Duo: A Signature Based Method to Batch-Analyze Functional Similarities of Proteins

**DOI:** 10.3389/fmicb.2021.698322

**Published:** 2021-08-12

**Authors:** Xiao Fei, Qiuchun Li, John Elmerdahl Olsen, Xinan Jiao

**Affiliations:** ^1^Key Laboratory of Prevention and Control of Biological Hazard Factors (Animal Origin) for Agri-food Safety and Quality, Ministry of Agriculture of China, Yangzhou University, Yangzhou, China; ^2^Jiangsu Key Lab of Zoonosis/Jiangsu Co-Innovation Center for Prevention and Control of Important Animal Infectious Diseases and Zoonoses, Yangzhou University, Yangzhou, China; ^3^Joint International Research Laboratory of Agriculture and Agri-Product Safety, Yangzhou University, Yangzhou, China; ^4^Department of Veterinary and Animal Sciences, Faculty of Health and Medical Sciences, University of Copenhagen, Copenhagen, Denmark

**Keywords:** biological signature, protein, bacteria, *Salmonella*, hidden Markov models

## Abstract

With the rapid advancement of sequencing technology, handling of large sequencing data to analyze for protein coding capacity and functionality of predicted proteins has become an urgent demand. There is a lack of simple and effective tools to functionally annotate large number of unknown proteins in a personalized and customized workflow. To address this, we developed Duo, which batch-analyze functional similarities of predicted proteins. Duo can screen query proteins with specific characteristics based on highly flexible and customizable reference inputs from the user. In the current study, Duo was applied to screen for virulence associated proteins in the genome-sequence of *Salmonella* Typhimurium. Based on the analysis, recommendation for choice of Seed_database in order to get a reasonable number of predicted proteins for further analysis, and recommendation for preparing a Reference_proteins set for Duo was given. Delta-bitscore analysis was shown to be useful tool to focus the follow-up on predicted proteins. A successful screen for virulence proteins in the bacterial genome-sequence was further performed in a selection of 32 pathogenic bacteria, documenting the ability of Duo to work on a broad collection of bacteria. We anticipate that Duo will be a useful auxiliary tool for personalized and customized protein function research in the future.

## Introduction

With the continuous evolution of next-generation sequencing (NGS) technologies, application of NGS methods for routine research is now possible at relatively low cost (Kircher and Kelso, 2010; Goodwin et al., 2016). As a result, customized ways to manage the constantly increasing amount of sequencing data has become urgent, particularly for functional categorization of proteins deduced from sequence data (Mitchell et al., 2019).

To address the demand of functional annotation of proteins, different methods have been developed for summarizing the functional similarity of proteins (known as “signatures”), such as hidden Markov models (HMMs)-based methods (HMMER^1^) (Eddy, 2011), and the position-specific scoring Matrices-based method PSI-BLAST (Altschul et al., 1997). In addition, sets of protein annotation databases have been established and are available for global data sharing, including InterPro, which integrates 14 different databases (Mitchell et al., 2019), and eggNOG, which is an HMM-based protein annotation database (Huerta-Cepas et al., 2019).

Advances in protein classification methods, coupled with various types of protein annotation databases, each focused on different types of proteins, have enabled a better understanding of unknown proteins. The most direct way is to check all annotation records manually, and then empirically select proteins of interest for further research. This method is selection free, allowing customizable selection of proteins for specific studies, however, it relies heavily on the experience and knowledge of the researcher and is unsuitable for high throughput screening. To facilitate function annotation, the annotation format has been standardized and classified according to different terminologies, such as Gene Ontology (GO),^2^ KEGG pathways^3^ (Kanehisa and Goto, 2000) and Clusters of Orthologous Groups (COG) (Tatusov et al., 1997). Moreover, annotation databases designed for specific research areas have been established, such as the Virulence Factor Database (VFDB^4^) (Liu et al., 2019) and the Comprehensive Antibiotic Resistance Database (CARD^5^) (Alcock et al., 2020). All these developments have promoted the efficiency of functional annotation. However, unlike the purely manual selection process, which can be highly customized, the protein filtering step depends on selecting predefined biological terms in the databases, and these are not always compatible with the specific research purposes. Therefore, it is necessary to develop a more flexible and customizable method for functional protein screening in large datasets.

In this paper, we present a new workflow named “Duo,” build to batch-analyze the functional similarities of proteins. Duo facilitates screening of query proteins with specific characteristics using freely available databased and customizable reference protein sequences defined by the user. As a case study, we applied Duo to screen for virulence associated proteins first in the genome sequence of *S.* Typhimurium and then more broadly in a selection of pathogenic bacteria using different, customizable input data. Duo is expected to become a valuable auxiliary tool for personalized and customized protein function prediction in the future.

## Materials and Methods

### Testing of Duo on Genomic and Meta-Data of *S.* Typhimurium

For Duo to work, one needs three components: a list of query proteins (the unknowns), a list of reference proteins with the characteristics one is searching for (in broad terms), defined by the user, and one or more reference databases. To evaluate the performance of Duo as a protein-function screening-utility and to discuss the influence of reference proteins and protein databases (in Duo termed Reference_proteins and Seed_database) on screening results, we established a case study to screen the protein coding sequences in the genome sequence of *S.* Typhimurium LT2 (in Duo termed Query_proteins) for proteins with virulence association. We downloaded the NCBI sequence record of *S.* Typhimurium LT2 (accession no. NC_003197, NC_003277) as the fixed input of Query_proteins. To obtain a list of experimentally verified virulence factors of *Salmonella*, we used the database VFDB.^6^ This database lists 167 out of 4548 protein coding sequences in LT2 as virulence associated proteins, which was used for calculating the success rate of screening for virulence associated proteins by Duo.

#### Experiment 1: Performance Comparison of Different Seed_Databases

In order to compare the influence of choice of Seed_databases on prediction of virulence associated proteins in *S.* Typhimurium, we used 14 publically available databases listed in InterPro as Seed_databases (Mitchell et al., 2019). In addition, we prepared two custom Seed_databases specially designed for this experiment, one listing proteins of *Escherichia coli* and *Salmonella* listed in the eggNOG database [15], and one listing proteins of Gamma-proteobacteria in the same database. Details of the Seed_databases are listed in Table 1. In this experiment, the protein sequence database named *E. coli*-vfdb (Table 2) was used as the input of Reference_proteins to obtain a set of proteins which were not identical to the Query_proteins set (*Salmonella*). The screening results based on different Seed_databases were summarized in “Interpro_all.Rtab,” then parsed by our custom R script “Compare_Seed_DB.R.”

**TABLE 1:** Overview of the Seed_databases applied in the study.

**Abbreviation**	**Full name**	**Biological entity**	**Signature method**	**Detail information or references**
GENE3D	CATH-Gene3D (v4.2.0)	Homologous Superfamilies	Profile HMMs	www.cathdb.info (Lewis et al., 2018; Sillitoe et al., 2019).
SUPERFAMILY	SUPERFAMILY (v1.75)	Homologous Superfamilies	Profile HMMs	supfam.org (Oates et al., 2015).
PFAM	Pfam (v33.1)	Domain, Families	Profile HMMs	pfam.xfam.org (El-Gebali et al., 2019).
SMART	SMART (v7.1)	Domain, Families	Profile HMMs	smart.embl.de (Letunic and Bork, 2018).
TIGRFAM	TIGRFAMS (v15.0)	Domain, Families	Profile HMMs	www.jcvi.org/research/tigrfams (Haft et al., 2013).
PIRSF	PIRSF (v3.10)	Domain, Families	Profile HMMs	proteininformationresource.org/pirsf/ (Nikolskaya et al., 2007).
SFLD	Structure–Function Linkage Database (v4)	Domain, Families	Profile HMMs	sfld.rbvi.ucsf.edu/archive/django/index.html (Akiva et al., 2014).
HAMAP	High-quality Automated and Manual Annotation of Proteins (v2020_01)	Domain, Families	Profiles	hamap.expasy.org/ (Pedruzzi et al., 2015).
PROSITE_PROFILES	PROSITE profiles (v2019_11)	Domain, Families	Profiles	prosite.expasy.org/ (Sigrist et al., 2013).
CDD	Conserved Domains and Protein Classification (v3.17)	Domain, Families	Profiles	www.ncbi.nlm.nih.gov/Structure/cdd/cdd.shtml (Marchler-Bauer et al., 2017).
PRINTS	PRINTS (v42.0)	Domain, Families	Profiles	www.bioinf.manchester.ac.uk/dbbrowser/PRINTS/ (Attwood et al., 2012).
PROSITE_PATTERNS	PROSITE patterns (v2019_11)	Features, sites	Patterns	prosite.expasy.org/ (Sigrist et al., 2013).
MOBIDB_LITE	MobiDB-lite (v2.0)	Intrinsic Disorder	Composition Prediction	Integrated in InterPro (Mitchell et al., 2019), annotation of long-range intrinsic disorder (provided by MobiDB-lite) (Piovesan et al., 2018).
COILS	coiled-coils	coiled coils	−	Integrated in InterPro (Mitchell et al., 2019), prediction of signal peptides, transmembrane regions and coiled-coils, via the SignalP, Phobius, TMHMM and Coils software packages.
CUSTOM_DB_1	−	Domain, Families	Profile HMMs	Profile HMMs for *Salmonella* and *Escherichia* proteins retrieved from the eggNOG database (Huerta-Cepas et al., 2019).
CUSTOM_DB_2	−	Domain, Families	Profile HMMs	Profile HMMs for Gammaproteobacterial proteins retrieved from the eggNOG database (Huerta-Cepas et al., 2019).

**TABLE 2:** Overview of Reference_proteins applied in this study.

**Name**	**Short name in figures**	**protein number**	**Detail information**	**others**
SetA-vfdb	SetA	3575	A core dataset includes genes associated with experimentally verified virulence factors only, downloaded from VFDB website (www.mgc.ac.cn/VFs/download.htm)	−
*E. coli*-vfdb	*E. coli*	293	A subset of SetA-vfdb only for the proteins from *E. coli*	*E. coli*: Gram-negative, Enterobacteriaceae family, Food poisoning
*Shigella*-vfdb	*Shigella*	104	A subset of SetA-vfdb only for the proteins from *Shigella*	Shigella: Gram-negative, Enterobacteriaceae family, Food poisoning
*C. jejuni*-vfdb	*C. jejuni*	128	A subset of SetA-vfdb only for the proteins from *C. jejuni*	*C. jejuni*: Gram-negative, Enterobacteriaceae family, Food poisoning
*S. aureus*-vfdb	*S. aureus*	95	A subset of SetA-vfdb only for the proteins from *S. aureus*	*S. aureus*: Gram-positive, Firmicutes Phylum, Food poisoning
*Clostridium*-vfdb	Clostridium	29	A subset of SetA-vfdb only for the proteins from *Clostridium*	*Clostridium*: Gram-positive, Firmicutes Phylum, Food poisoning
*Salmonella*-vfdb	*Salmonella*	130	A subset of SetA-vfdb only for the proteins from *Salmonella*	*Salmonella*: Gram-negative, Enterobacteriaceae family, Food poisoning
Non-*Salmonella*-vfdb	Non-*Salmonella*	3445	A subset of SetA-vfdb excluding the proteins from *Salmonella*	−

In order to observe the delta-bitscore (Wheeler et al., 2016) (protein functional similarity index) distribution between experimentally verified and unverified virulence encoding proteins predicted in the screen, delta-bitscore results were summarized in “cross_result.Rtab.” Subsequently, it was parsed with our custom R script “cross_analysis.R.” Briefly, the delta-bitscore results observed with different Seed_databases were recorded. If a predicted protein was assigned several bitscores by a Seed_database, which could happen if the database predicted function based on different signatures, the lowest delta-bitscore was selected to represent the delta-bitscore for the virulence factors.

#### Experiment 2: Performance Comparison of Different Reference_Proteins Sets

To compare different sets of Reference_proteins on the prediction of virulence associated proteins, we used eight different sets of Reference_proteins (Table 2). In this experiment, 14 available public Seed_databases (Table 1) were applied in combination with each of the eight Reference_proteins. The screening results with different Reference_proteins were summarized in “Interpro_all.Rtab.combine,” then parsed by our custom R script “Compare_reference_proteins.R.”

### Testing of Duo on a Broad Selection of Pathogenic Bacteria

To validate that Duo can be used on a wide variety types of bacteria, we further use Duo to screen for virulence associated proteins in a broad selection of pathogenic bacteria (32 common bacterial pathogens). Similar to the former case study of *Salmonella*, for every screening of a single bacterial species, one needs to prepare three inputs: Query_proteins, Reference_proteins, and Seed_database. Briefly, in the preparation of Query_proteins, we selected a representative whole genome sequence in the target species (same as the representative of that species listed in VFDB) and extracted all the coding sequences. In the selection of Reference_proteins, firstly, SetA-vfdb (Table 2), a core dataset including bacterial genes associated with experimentally verified virulence factors only, was selected as the basis for selection of “Reference_proteins.” To avoid that query proteins were identical to reference proteins, the final “Reference_proteins” for the target species was constructed by excluding the subset of SetA-vfdb proteins from the target species itself (e.g., the Reference_proteins for *Salmonella* consisted of the virulence proteins in the VFDB database, but excluding the proteins from *Salmonella*). The Seed_databases were retrieved from the eggNOG database^7^ according to the taxonomic grouping of target species (e.g., *Salmonella* belong to the Class Gammaproteobacteria, and hence the Profile HMMs of Gammaproteobacteria in the eggNOG database was used as the Seed_database for this species). A detailed description of inputs per species are summarized in Supplementary Table 1.

## Results and Discussion

### Overview of the Duo Workflow

**FIGURE 1:**
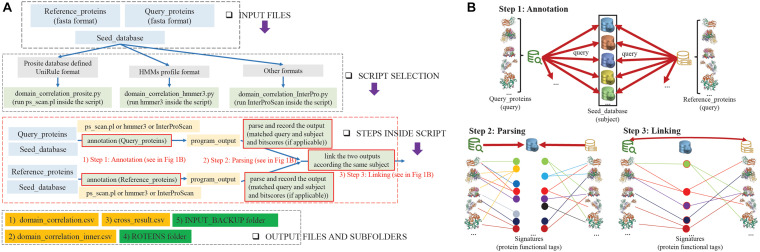
Overview of Duo workflow. **(A)** Flowcharts of the steps in the application of Duo. INPUT FILES: Before the analysis, user needs to prepare three files containing the protein coding sequences that needs to be analyzed (Query_proteins), a set of sequences that are known to encode proteins of the wanted quality (Reference_proteins), and a database to search in (Seed_database). SCRIPT SELECTION: Seed_databases are in different formats, and to make it possible to use Duo broadly, it contains different python scripts to be selected depending on the format of Seed_database. STEPS INSIDE SCRIPT: The data processing steps of Duo (further descripted in Figure) consists of three general steps. (1) ANNOTATION: the Query_proteins and Reference_proteins are annotated separately according to the Seed_database. (2) PARSING: The annotation results are parsed and recorded. (3) LINKING: The parsed annotation results from Query_proteins and Reference_proteins are linked. OUTPUT FILES AND SUBFOLDERS: Based on the analysis, Duo constructs three output files. (1) Domain_correlation.csv gives details of all the correlation records (parsed annotation results from Query_proteins and Reference_proteins). (2) Domain_correlation_inner.csv gives details of the correlation records shared between the linked Query_proteins and Reference_proteins. (3) Cross_result.csv records the delta-bitscores between the linked Query_proteins and Reference_proteins. The output further contains two sub-folders (4) PROTEINS, which gives records of the functional similar proteins among Query_proteins and Reference_proteins and (5) INPUT_BACKUP, which backs up the original files of Query_proteins and Reference_proteins. **(B)** A schematic illustration of the three steps in Duo analysis. Step 1: Separately, Query_protein and Reference_proteins are used as query inputs to query the same Seed_database(s) (subject input) by suitable programs (ps_scan.pl or hmmer3 or InterProScan). Step 2: The matched protein functional tags (signatures) for Query_proteins or Reference_proteins are recorded together with the related bitscore(s) (if applicable). Step 3: Based on the outputs from the previous step, the parsed records from Query_proteins and Reference_proteins are linked if they contain the same functional tag, and if applicable, the absolute value of the bitscores differences between the linked Query_proteins and Reference_proteins (delta-bitscore) is calculated.

Figure 1 shows an overview of the Duo workflow and details the steps in the application of Duo. The Duo workflow contains three input parts, which are defined by users for specific research purposes. We named these three parts Query_proteins, Reference_proteins, and Seed_database. Both Query_proteins and Reference_proteins are protein sequence files in fasta format. Query_proteins are the candidate proteins of interest for the user. Seed_database are database(s) of different types of protein signatures, whose biological entities will be used as the correlation point(s) between Query_proteins and Reference_proteins, e.g., hidden Markov models (HMMs) method-based databases (Pfam (El-Gebali et al., 2019), TIGRFAM (Haft et al., 2013), and SMART (Letunic and Bork, 2018)) or profile method-based databases (HAMAP (Pedruzzi et al., 2015), Prosite (Sigrist et al., 2013), and CDD (Marchler-Bauer et al., 2017)). Duo has been designed to work with different formats of Seed_databases, and as shown in Figure 1A, before analysis can begin, the user needs to choose one of the three python scripts who are designed to handling with different format of Seed_database. After assigning these three inputs, Duo will automatically query the Query_proteins and Reference_proteins against Seed_databases, and the matched point(s) (biological entity term(s) between Query_proteins/Reference_proteins and Seed_database) will be recorded for further analysis (Figure 1B Steps 1 and 2). Next, Duo will associate the Query_proteins with Reference_proteins according the same matched point(s) (Figure 1B Step 3).

The Duo workflow creates three output files and two subfolders detailing the correlations among Query_proteins, Reference_proteins, and Seed_database. “Domain_correlation.csv” record details of all the correlation records (parsed annotation results from Query_proteins and Reference_proteins); “Domain_correlation_inner.csv” only details the correlation records shared between Query_proteins and Reference_proteins; and “cross_result.csv” details the delta-bitscore (Wheeler et al., 2016) (protein functional similarity score) records between Query_proteins and Reference_proteins. Finally, the functional similar proteins among Query_proteins and Reference_proteins are stored in a file in the “PROTEINS” folder and the original files of Query_proteins and Reference_proteins back up in “INPUT_BACKUP” folder. Full details of the usage and outputs are provided on project home page of Duo.^8^

### The Influence of Seed_Databases on Functional Protein Prediction

**FIGURE 2:**
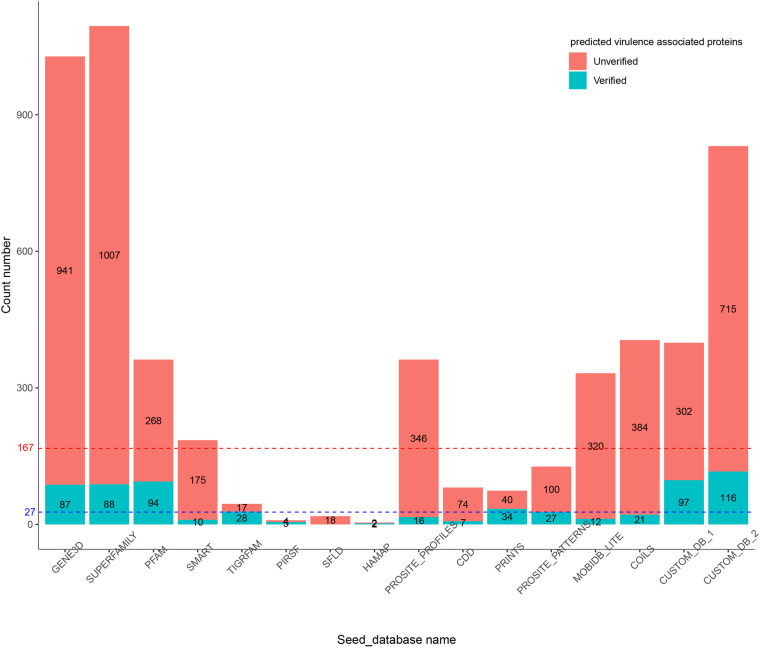
Number of predicted virulence associated proteins in *S.* Typhimurium by Duo based on different Seed_databases. The blue dotted line indicates the medium number of predicted protein. The red dotted line indicates the total number of experimentally verified virulence associated proteins in *S*. Typhimurium LT2.

**FIGURE 3:**
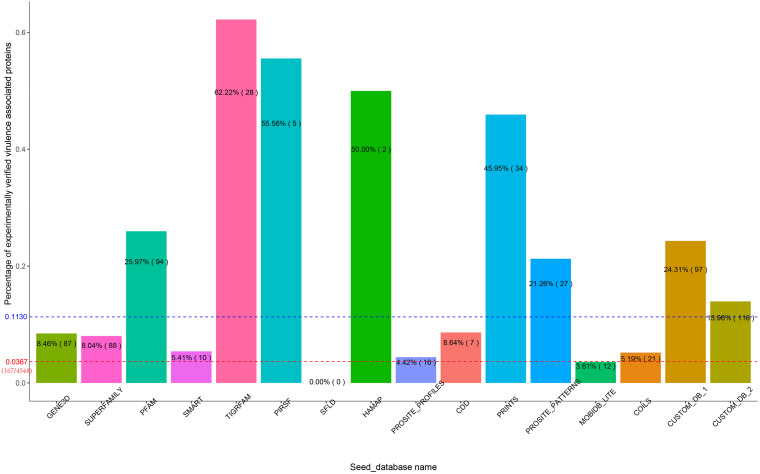
Percent of experimentally verified *S.* Typhimurium virulence associated proteins among the predicted proteins with each Seed_database. The blue dotted line indicates the medium percentage among the Seed-database. For comparison, the red dotted line indicates the percentage of experimentally verified virulence proteins in *S*. Typhimurium out of all proteins encoded from this bacterium.

In the first experiment, Duo was applied to screen for *S.* Typhimurium virulence associated proteins based on the experimentally verified virulence factors from *E. coli*. *S.* Typhimurium was chosen as the study object, because the pathogenesis is well described and a high number of virulence factors of different types have been identified and verified experimentally. The screening was performed with different Seed_databases and results are summarized in Figures 2, 3. GENE3D, SUPERFAMILY, and CUSTOM_DB_2 databases ranked in the top three according to the number of predicted virulence associated proteins, and HAMAP, PIRSF, and SFLD databases were in the bottom (Figure 2). Unlike other Seed_databases, the biological entity in GENE3D and SUPERFAMILY is Homologous Super-families (Table 1), which is a more general entity than that of biological entities Domain and Family (Mitchell et al., 2019). Similarly, compared with CUSTOME_DB_1 (Profile HMMs for *Salmonella* and *Escherichia* proteins retrieved from the eggNOG database), CUSTOME_DB_2 is a more general profile (Profile HMMs for Gamma-proteobacteria proteins retrieved from the eggNOG database). These results indicate that using a more general set of criteria for Seed_database in Duo results in a higher number of predicted proteins of the desired type. Both PIRSF and SFLD focus on protein clustering based on apparent evolutionary relationships between proteins (Nikolskaya et al., 2007; Akiva et al., 2014). Even though we attempted to predict proteins with similar characteristics (i.e., virulence associated proteins) based on query proteins and reference proteins from closely related bacterial species (*Salmonella* and *E. coli*), using these Seed_databases resulted in a low number of hits, indicating that such databases are less suited for this purpose. TIGRFAM focuses on the annotation of prokaryotic proteins (Haft et al., 2013), and it thus should be suitable for the study of the organisms used in this experiment (i.e., bacteria). Notably, even though the total number of predicted virulence associated proteins was not high using this database (Figure 2), the number of experimentally verified virulence associated proteins was above the medium level, and with 62% of the predicted ones, it showed the highest rate of experimentally verified virulence associated proteins among the databases tested (Figure 3).

To further illustrate the importance of Seed_database when screening for proteins with specific characteristics, we built two customized Seed_databases (Table 1) containing protein sequences of only the input species (*Salmonella*) and the closely related bacterium *E. coli*. The results showed that both of the two custom Seed_databases exhibited good performance with an above medium number of experimentally verified proteins (Figure 2) and at the same time an above medium number of verification rate of the total predicted proteins (Figure 3).

In summary, the results of our first experiment clearly showed the influence of the choice of Seed_database on the performance of Duo for functional protein screening. A general recommendation for choice of Seed_database in order to get a reasonable number of predicted proteins for further analysis appears to combine a broad classification scale (Super family, etc.) with a database which is aligned with the type of bacteria under research, however, as shown from the better performance of CUSTOM_DB_2 over CUSTOM_DB_1, not limited to the narrow group of bacteria investigated. This is because proteins are unlikely to be annotated with unknown function, if it is closely related to another protein in the same species. It should be noted that making custom made databases may not always be straight forward, as in the current example.

### The Influence of Reference_Proteins on Functional Protein Prediction

**FIGURE 4:**
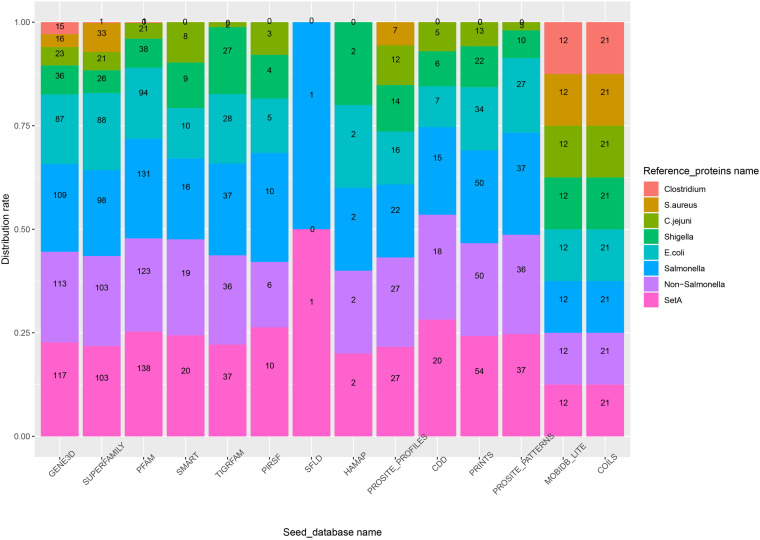
The number of experimentally verified virulence associated proteins in *S.* Typhimurium (numbers inside bars is verified numbers) among the predicted proteins depending on the source of the reference proteins (color of bars) and the Seed_database used. The scale on the Y-axis shows the accumulated percentage of verified numbers by that database when the results from different Reference_protein group were stacked on top of each other.

In experiment 2, Duo was applied to screen *S.* Typhimurium LT2 for virulence associated proteins using different sets of Reference_proteins, consisting of experimentally verified virulence proteins from of different sources (Table 2). The result in Figure 4 showed that using setA-vfdb, Non-*Salmonella*-vfdb or *Salmonella*-vfdb Reference_proteins resulted in prediction of a similar number of experimentally verified virulence associated protein, and the numbers of proteins identified were higher than with other sets. It is not surprising that the *Salmonella*-vfdb and SetA-vfdb groups resulted in relatively high numbers of experimentally verified virulence associated protein, as they contain virulence proteins of same species as the Query_proteins (*S.* Typhimurium LT2). Interestingly, the verified numbers were similar between the SetA-vfdb database and the Non-*Salmonella*-vfdb database (A subset of SetA-vfdb excluding the proteins from *Salmonella*). This result showed that the Duo workflow is suitable for identification of functionally similar proteins across species. The composition of Reference_proteins could be a key factor for the precision of the screen. One notable example is the difference in the rate of experimentally verified proteins among predicted virulence proteins when using *Salmonella*-vfdb compared to Non-*Salmonella*-vfdb as Reference_proteins. The two screens showed similar number of experimentally verified proteins (Figure 4) but the rate was much higher using the *Salmonella*-vfdb proteins (Supplementary Figure 1). Non-*Salmonella*-vfdb contains 3455 reference virulence proteins sourced from different species. While this multiple source composition can introduce more biological signatures, thereby improving screening ability across different evolutionary backgrounds, this inevitably introduces more non-specific biological signatures, which may increase the number of false positive predictions among the screened proteins.

To analyze this, we prepared five Reference_protein databases sourced from different public health relevant bacteria (Table 2). This included *Salmonella* (the target), and *E. coli*, *Shigella*, and *Campylobacter jejuni*, all Gram-negative bacteria classified as Gammaproteobacteria. For convenience, we named these Reference_protein databases the Gram-negative sets. In addition, we build Reference_protein databases for the Gram-positive bacteria *Staphylococcus aureus* and *Clostridium*, which belong to Firmicutes Phylum. We named these two databases the Gram-positive sets. As shown in Figure 4, the experimentally verified number of virulence associated proteins was always higher when using the Gram-negative sets as reference compared to the Gram-positive sets. This result indicated that the evolutionary relationship between query and reference proteins influence the outcome when using Duo for functional protein screening; the closer the evolutionary distance the more specific the outcome will be. In addition, compared with the non-*Salmonella*-vfdb database, the *E.coli*-vfdb and *Shigella*-vfdb databases showed better screening credibility (higher verification rate, Supplementary Figure 1). Based on all these observations, the general recommendation for preparing a Reference_proteins set for Duo is to make a custom Reference_proteins set which spans across species, but the evolutionary distance between the “Query-species” and the species included in the Reference_proteins should not be too distant, and one should avoid Reference_proteins sets which mix too many attributes, as this may reduce the precision of predictions.

### Delta-Bitscore, an Auxiliary Reference Score for Evaluation of Functional Protein Screening

**FIGURE 5:**
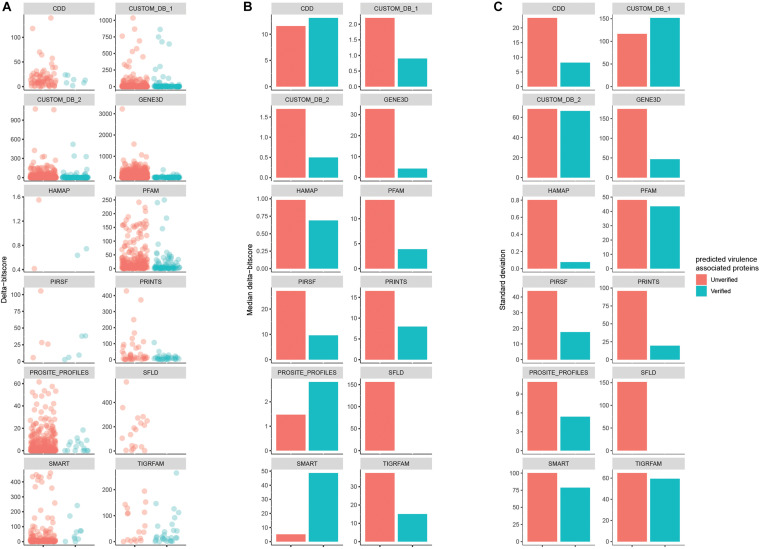
Comparison of the delta-bitscore of predicted proteins depending on Seed_database divided into experimentally verified virulence associated proteins (red dots) and unverified ones (blue dots) **(A)**. The median of delta-bitscore results on the data are shown in **(B)** and the standard deviation of the results in **(C)**.

The delta-bitscore was first introduced as part of studies of *Salmonella* adaptation (Kingsley et al., 2013) and is a credible index to rank the functional similarity of orthologous genes (Clifford et al., 2004; Shihab et al., 2013; Liu et al., 2015; Wheeler et al., 2016). It is the absolute value of the bitscores differences between Query_protein and Reference_protein with the same matched biological signature. Duo calculates the delta-bitscore for every matched record between query and reference proteins. This is illustrated in Figure 5 based on data from experiment 2. The databases COLIS, MOBIDB_LITE, PROSITE_PATTERNS, and SUPERFAMILY are incompatible with delta-bitscore measures, so no delta-bitscore results could be obtained for these. The results showed that for most of the different Seed_databases (9/12), the median delta-bitscore was lower for experimentally verified virulence factors than for the unverified ones. This corresponds well to the fact that the lower the delta-bitscore, the higher the functional similarity between the query and reference protein (Clifford et al., 2004; Shihab et al., 2013; Liu et al., 2015; Wheeler et al., 2016). This result implies that delta-bitscore is a good tool to evaluate the precision of one’s screen. According to the results in 6A, the delta-bitscore generally appeared more uniform among the verified proteins. In concordance with this, the standard deviation of delta-bitscores was lower in the experimentally verified virulence associated proteins with 11 out of the 12 databases compared to the unverified ones. A low standard deviation indicates that the values tend to be close to the mean (Pearson and Henrici, 1997). This indicated that filtering the predicted proteins based on a sub-range of delta-bitscore may improve the precision of the functional protein prediction.

In summary, the delta-bitscore analysis on data from experiment 2 indicated the usability of this score in functional protein-prediction as a tool to focus the follow-up on predicted proteins with low delta-bitscore, and if the number of predicted target proteins is large, to use delta-bitscore in further filtering to concentrate on a fixed sub-range of delta-bitscore.

### Application of Duo on a Broad Spectrum of Bacteria

Duo is designed as an auxiliary tool to facilitate the biological signature correlations among proteins. Theoretically, Duo can be used to screen proteins with specific characteristics on any organisms. In order to practically validate the feasibility of using Duo on a broad spectrum of bacteria, we selected one strain of 32 bacterial genera, and screened the genomes for virulence associate proteins. The total protein-encoding sequence number and the experimentally verified virulence associated proteins among them were counted as summarized in Table 3. The results showed that for 31 out of 32 of the tested strains, the rate of verified virulence proteins increased by the screening. Simultaneously, for all but six of these 31 tested strains, the verified pass rate was over 70%. These results indicates that Duo mainly eliminates the non-virulence associate proteins (reflected by increased verified rate after screen) and contains the virulence associate ones (reflected by high verified pass rate after screen). The result supports that Duo works well on a broad spectrum of bacteria. It is worth noting that event though Duo increased the verified rate in most bacteria, it was still at a relative low level (less than 10%). This is because we applied Reference_proteins with multiple source composition, which may reduce the screening specificity, as discussed in the section named “The influence of Reference_proteins on functional protein prediction.” In most cases, users will have specific background knowledge about their research target, and thus they can use more specific and customized Reference_proteins input to achieve better predictions.

**TABLE 3:** Number of Duo-predicted and verified virulence associated proteins in genomes of selected pathogenic bacteria and its experimentally verified virulence associate ones before and after Duo screening.

**Target genera**	**Before screen**			**After screen**			**Verified rate change^*f*^**	**Verified pass rate^*g*^**
	**Verified protein number^a^**	**Total protein number^b^**	**Verified rate^c^**	**Verified protein number^d^**	**Total protein number^e^**	**Verified rate^c^**		
*Acinetobacter*	80	3667	2.18%	70	1737	4.03%	+1.85%	87.50%
*Aeromonas*	165	4122	4.00%	153	2123	7.21%	+3.20%	92.73%
*Anaplasma*	29	1352	2.14%	19	322	5.90%	+3.76%	65.52%
*Bacillus*	32	5330	0.60%	25	2467	1.01%	+0.41%	78.13%
*Bartonella*	55	1612	3.41%	38	605	6.28%	+2.87%	69.09%
*Bordetella*	112	3806	2.94%	97	2060	4.71%	+1.77%	86.61%
*Brucella*	52	3198	1.63%	50	1553	3.22%	+1.59%	96.15%
*Burkholderia*	146	5855	2.49%	130	3032	4.29%	+1.79%	89.04%
*Campylobacter*	130	1643	7.91%	120	780	15.38%	+7.47%	92.31%
*Chlamydia*	46	886	5.19%	21	241	8.71%	+3.52%	45.65%
*Clostridium*	17	3897	0.44%	14	1953	0.72%	+0.28%	82.35%
*Corynebacterium*	33	2320	1.42%	32	951	3.36%	+1.94%	96.97%
*Coxiella*	151	1850	8.16%	46	687	6.70%	-1.47%	30.46%
*Enterococcus*	35	3247	1.08%	28	1402	2.00%	+0.92%	80.00%
*Escherichia*	125	4685	2.67%	114	2153	5.29%	+2.63%	91.20%
*Francisella*	127	1804	7.04%	91	674	13.50%	+6.46%	71.65%
*Haemophilus*	85	1709	4.97%	70	699	10.01%	+5.04%	82.35%
*Helicobacter*	87	1566	5.56%	47	588	7.99%	+2.44%	54.02%
*Klebsiella*	124	5244	2.36%	116	2524	4.60%	+2.23%	93.55%
*Legionella*	126	2942	4.28%	92	1308	7.03%	+2.75%	73.02%
*Listeria*	45	2867	1.57%	37	1422	2.60%	+1.03%	82.22%
*Mycobacterium*	253	4031	6.28%	186	1966	9.46%	+3.18%	73.52%
*Mycoplasma*	10	688	1.45%	2	124	1.61%	+0.16%	20.00%
*Neisseria*	89	2063	4.31%	73	700	10.43%	+6.11%	82.02%
*Pseudomonas*	252	5571	4.52%	212	2953	7.18%	+2.66%	84.13%
*Rickettsia*	28	838	3.34%	26	324	8.02%	+4.68%	92.86%
*Salmonella*	167	4548	3.67%	140	2089	6.70%	+3.03%	83.83%
*Shigella*	92	4698	1.96%	81	1978	4.10%	+2.14%	88.04%
*Staphylococcus*	97	2695	3.60%	73	1221	5.98%	+2.38%	75.26%
*Streptococcus*	41	1865	2.20%	27	808	3.34%	+1.14%	65.85%
*Vibrio*	166	3828	4.34%	151	1705	8.86%	+4.52%	90.96%
*Yersinia*	150	4217	3.56%	140	2099	6.67%	+3.11%	93.33%

*^*a*^**Verified protein number** indicates the number of experimentally verified virulence associated proteins in the genera.*

*^*b*^**Total protein number** is the number of protein coding sequences in the genome corresponding to the number of query proteins for Duo.*

*^*c*^**Verified rate** is the percentage of experimentally verified virulence associate proteins among the total proteins (Verified rate = Verified protein number/Total protein number × 100%).*

*^*d*^The number of experimentally verified virulence associated proteins among the virulence associated proteins predicted by Duo.*

*^*e*^The predicted number of virulence associated proteins by Duo.*

*^*f*^**Verified rate change** is the change in verified rates before and after screen (Verified rate change = verified rate (after screen) – verified rate (before screen)). “+” means the verified rate has increased after screen, and ‘-’ means the verified rate has dropped after screen.*

*^*g*^**Verified pass rate** is the rate of experimentally verified virulence associate proteins among the predicted virulence proteins from Duo (Verified pass rate = Verified protein number (after screen)/Verified protein number (before screen) × 100%).*

## Conclusion

With the rapid advancement of sequencing technology (Kircher and Kelso, 2010; Goodwin et al., 2016), handling the enormous and constantly increasing amount of protein-encoding sequence data has become one of the most urgent demands among the scientific community (Mitchell et al., 2019). In this study, we present a biological signature-based method to batch-analyze the functional similarities of proteins. We have named the method Duo. Duo provides an easy and effective way for batch scoring of the functional similarity between query and reference proteins. As a key utility, Duo allows to screen proteins with unknown function for specific characteristics using free and customizable reference protein sequence inputs defined by the user. We anticipate that Duo will be a useful auxiliary tool for personalized and customized protein function research.

## Data Availability Statement

Publicly available datasets were analyzed in this study. This data can be found here: https://github.com/china-fix/Duo.

## Author Contributions

XF designed and implemented the workflow and carried out the majority of the analyses with input from JO and QL. XF and JO wrote the manuscript with input from XJ and QL. JO and XJ guided the research with input from QL. All authors contributed to the article and approved the submitted version.

## Conflict of Interest

The authors declare that the research was conducted in the absence of any commercial or financial relationships that could be construed as a potential conflict of interest.

## Publisher’s Note

All claims expressed in this article are solely those of the authors and do not necessarily represent those of their affiliated organizations, or those of the publisher, the editors and the reviewers. Any product that may be evaluated in this article, or claim that may be made by its manufacturer, is not guaranteed or endorsed by the publisher.

## Footnotes

1
http://hmmer.org/

2
http://geneontology.org/

3
https://www.kegg.jp/kegg/

4
http://www.mgc.ac.cn/VFs/

5
https://card.mcmaster.ca/

6
http://www.mgc.ac.cn/VFs/download.htm

7
http://eggnog5.embl.de/#/app/downloads

8
https://github.com/china-fix/Duo
