# Supplementary material for: Duo: A Signature Based Method to Batch-Analyze Functional Similarities of Proteins
Source: Front Microbiol. 2021 Aug 12;12:698322. doi: 10.3389/fmicb.2021.698322 (PMC8406696; doi:10.3389/fmicb.2021.698322)
Supplement: Supplementary file 1 [file Data_Sheet_1.docx]

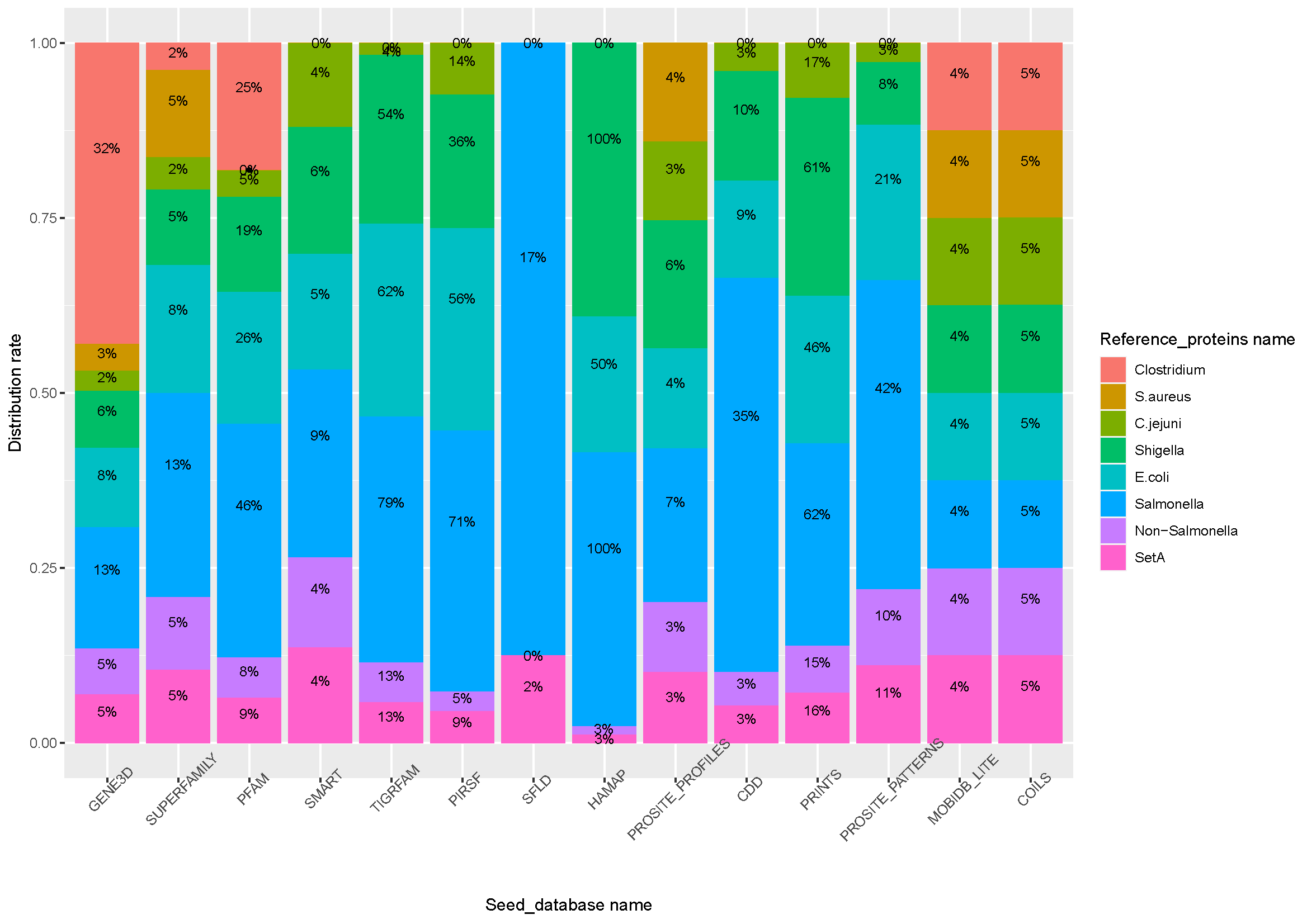


**Supplementary Figure S1.** The percent of experimentally verified virulence associated proteins in *Salmonella* Typhimurium (percent inside bars is verified rate) among the predicted proteins depending on the source of the reference proteins (colour of bars) and the Seed_database used. The scale on the Y-axis shows the accumulated verified rates by that database when the results from different Reference_protein group were stacked on top of each other.

**Supplementary Table S1.** Detailed explanation of all the inputs applied in the testing of Duo on a broad selection of pathogenic bacteria

| Target genera | Query_proteins | | Reference_proteins | | Seed_databases | |
| --- | --- | --- | --- | --- | --- | --- |
|  | **download URL:** [**https://github.com/china-fix/Duo/tree/main/toy_data/Query_proteins**](https://github.com/china-fix/Duo/tree/main/toy_data/Query_proteins) | | **download URL:** [**https://github.com/china-fix/Duo/tree/main/toy_data/Reference_proteins**](https://github.com/china-fix/Duo/tree/main/toy_data/Reference_proteins) | | **download URL:** [**http://eggnog5.embl.de/#/app/downloads**](http://eggnog5.embl.de/#/app/downloads) | |
|  | **File name** | **Technical information (representative strain’s ID)** | **File name** | **Technical information** | **File name** | **Technical information (the Class of target genera)** |
| *Acinetobacter* | Acinetobacter.fa.p | *A. baumannii* ACICU chromosome NC_010611 (3904116 bp) | VFDB_setA_NO_Acinetobacter.fas | A subset of SetA-vfdb excluding the proteins from *Acinetobacter* | Gammaproteobacteria | Gammaproteobacteria |
| *Aeromonas* | Aeromonas.fa.p | *A. hydrophila* subsp. hydrophila ATCC 7966 chromosome NC_008570 (4744448 bp) | VFDB_setA_NO_Aeromonas.fas | A subset of SetA-vfdb excluding the proteins from *Aeromonas* | Gammaproteobacteria | Gammaproteobacteria |
| *Anaplasma* | Anaplasma.fa.p | *A. phagocytophilum* HZ chromosome NC_007797 (1471282 bp) | VFDB_setA_NO_Anaplasma.fas | A subset of SetA-vfdb excluding the proteins from *Anaplasma* | Alphaproteobacteria | Alphaproteobacteria |
| *Bacillus* | Bacillus.fa.p | *B. anthracis str. Ames* (pXO1- pXO2-) chromosome NC_003997 (5227293 bp) | VFDB_setA_NO_Bacillus.fas | A subset of SetA-vfdb excluding the proteins from *Bacillus* | Firmicutes | Firmicutes |
| *Bartonella* | Bartonella.fa.p | *B. henselae str.* Houston-1 chromosome NC_005956 (1931047 bp) | VFDB_setA_NO_Bartonella.fas | A subset of SetA-vfdb excluding the proteins from *Bartonella* | Alphaproteobacteria | Alphaproteobacteria |
| *Bordetella* | Bordetella.fa.p | *B. pertussis Tohama I* chromosome NC_002929 (4086189 bp) | VFDB_setA_NO_Bordetella.fas | A subset of SetA-vfdb excluding the proteins from *Bordetella* | Betaproteobacteria | Betaproteobacteria |
| *Brucella* | Brucella.fa.p | *B. melitensis bv. 1 str.* 16M chromosome I NC_003317 (2117144 bp) chromosome II NC_003318 (1177787 bp) | VFDB_setA_NO_Brucella.fas | A subset of SetA-vfdb excluding the proteins from *Brucella* | Alphaproteobacteria | Alphaproteobacteria |
| *Burkholderia* | Burkholderia.fa.p | *B. pseudomallei* K96243 chromosome I NC_006350 (4074542 bp) chromosome II NC_006351 (3173005 bp) | VFDB_setA_NO_Burkholderia.fas | A subset of SetA-vfdb excluding the proteins from *Burkholderia* | Betaproteobacteria | Betaproteobacteria |
| *Campylobacter* | Campylobacter.fa.p | *C. jejuni subsp. jejuni* NCTC 11168 chromosome NC_002163 (1641481 bp) | VFDB_setA_NO_Campylobacter.fas | A subset of SetA-vfdb excluding the proteins from *Campylobacter* | delta/epsilon subdivisions | delta/epsilon subdivisions |
| *Chlamydia* | Chlamydia.fa.p | *C. trachomatis* D/UW-3/CX (serovar D) chromosome NC_000117 (1042519 bp) | VFDB_setA_NO_Chlamydia.fas | A subset of SetA-vfdb excluding the proteins from *Chlamydia* | Chlamydiae | Chlamydiae |
| *Clostridium* | Clostridium.fa.p | *C. difficile* 630 chromosome NC_009089 (4290252 bp) | VFDB_setA_NO_Clostridium.fas | A subset of SetA-vfdb excluding the proteins from *Clostridium* | Firmicutes | Firmicutes |
| *Corynebacterium* | Corynebacterium.fa.p | *C. diphtheriae* NCTC 13129 (biotype gravis) chromosome NC_002935 (2488635 bp) | VFDB_setA_NO_Corynebacterium.fas | A subset of SetA-vfdb excluding the proteins from *Corynebacterium* | Actinobacteria | Actinobacteria |
| *Coxiella* | Coxiella.fa.p | *C. burnetii* RSA 493 chromosome NC_002971 (1995281 bp) pQpH1 NC_004704 (37393 bp) | VFDB_setA_NO_Coxiella.fas | A subset of SetA-vfdb excluding the proteins from *Coxiella* | Gammaproteobacteria | Gammaproteobacteria |
| *Enterococcus* | Enterococcus.fa.p | *E. faecalis* V583 chromosome NC_004668 (3218031 bp) pTEF1 NC_004669 (66320 bp) pTEF2 NC_004671 (57660 bp) | VFDB_setA_NO_Enterococcus.fas | A subset of SetA-vfdb excluding the proteins from *Enterococcus* | Firmicutes | Firmicutes |
| *Escherichia* | Escherichia.fa.p | *E. coli* 536 (UPEC) chromosome NC_008253 (4938920 bp) | VFDB_setA_NO_Escherichia.fas | A subset of SetA-vfdb excluding the proteins from *Escherichia* | Gammaproteobacteria | Gammaproteobacteria |
| *Francisella* | Francisella.fa.p | *F. tularensis subsp. tularensis* SCHU S4 chromosome NC_006570 (1892775 bp) | VFDB_setA_NO_Francisella.fas | A subset of SetA-vfdb excluding the proteins from *Francisella* | Gammaproteobacteria | Gammaproteobacteria |
| *Haemophilus* | Haemophilus.fa.p | *H. influenzae* Rd KW20 (serotype d) chromosome NC_000907 (1830138 bp) | VFDB_setA_NO_Haemophilus.fas | A subset of SetA-vfdb excluding the proteins from *Haemophilus* | Gammaproteobacteria | Gammaproteobacteria |
| *Helicobacter* | Helicobacter.fa.p | *H. pylori* 26695 chromosome NC_000915 (1667867 bp) | VFDB_setA_NO_Helicobacter.fas | A subset of SetA-vfdb excluding the proteins from *Helicobacter* | delta/epsilon subdivisions | delta/epsilon subdivisions |
| *Klebsiella* | Klebsiella.fa.p | *K. pneumoniae subsp. pneumoniae* NTUH-K2044 chromosome NC_012731 (5248520 bp) pK2044 NC_006625 (224152 bp) | VFDB_setA_NO_Klebsiella.fas | A subset of SetA-vfdb excluding the proteins from *Klebsiella* | Gammaproteobacteria | Gammaproteobacteria |
| *Legionella* | Legionella.fa.p | *L. pneumophila subsp. pneumophila str.* Philadelphia 1 chromosome NC_002942 (3397754 bp) | VFDB_setA_NO_Legionella.fas | A subset of SetA-vfdb excluding the proteins from *Legionella* | Gammaproteobacteria | Gammaproteobacteria |
| *Listeria* | Listeria.fa.p | *L. monocytogenes EGD-e* (serovar 1/2a) chromosome NC_003210 (2944528 bp) | VFDB_setA_NO_Listeria.fas | A subset of SetA-vfdb excluding the proteins from *Listeria* | Firmicutes | Firmicutes |
| *Mycobacterium* | Mycobacterium.fa.p | *M. tuberculosis* H37Rv chromosome NC_000962 (4411532 bp) | VFDB_setA_NO_Mycobacterium.fas | A subset of SetA-vfdb excluding the proteins from *Mycobacterium* | Actinobacteria | Actinobacteria |
| *Mycoplasma* | Mycoplasma.fa.p | *M. pneumoniae* M129 chromosome NC_000912 (816394 bp) | VFDB_setA_NO_Mycoplasma.fas | A subset of SetA-vfdb excluding the proteins from *Mycoplasma* | Tenericutes | Tenericutes |
| *Neisseria* | Neisseria.fa.p | *N. meningitidis* MC58 (serogroup B) chromosome NC_003112 (2272360 bp) | VFDB_setA_NO_Neisseria.fas | A subset of SetA-vfdb excluding the proteins from *Neisseria* | Betaproteobacteria | Betaproteobacteria |
| *Pseudomonas* | Pseudomonas.fa.p | *P. aeruginosa* PAO1 chromosome NC_002516 (6264404 bp) | VFDB_setA_NO_Pseudomonas.fas | A subset of SetA-vfdb excluding the proteins from *Pseudomonas* | Gammaproteobacteria | Gammaproteobacteria |
| *Rickettsia* | Rickettsia.fa.p | *R. typhi str.* Wilmington chromosome NC_006142 (1111496 bp) | VFDB_setA_NO_Rickettsia.fas | A subset of SetA-vfdb excluding the proteins from *Rickettsia* | Alphaproteobacteria | Alphaproteobacteria |
| *Salmonella* | Salmonella.fa.p | *S. enterica subsp. enterica* serovar Typhimurium str. LT2 chromosome NC_003197 (4857432 bp) pSLT NC_003277 (93939 bp) | VFDB_setA_NO_Salmonella.fas | A subset of SetA-vfdb excluding the proteins from *Salmonella* | Gammaproteobacteria | Gammaproteobacteria |
| *Shigella* | Shigella.fa.p | *S. flexneri* 2a str. 301 (serotype 2a) chromosome NC_004337 (4607203 bp) pCP301 NC_004851 (221618 bp) | VFDB_setA_NO_Shigella.fas | A subset of SetA-vfdb excluding the proteins from *Shigella* | Gammaproteobacteria | Gammaproteobacteria |
| *Staphylococcus* | Staphylococcus.fa.p | *S. aureus subsp. aureus* MW2 chromosome NC_003923 (2820462 bp) | VFDB_setA_NO_Staphylococcus.fas | A subset of SetA-vfdb excluding the proteins from *Staphylococcus* | Firmicutes | Firmicutes |
| *Streptococcus* | Streptococcus.fa.p | *S. pyogenes* MGAS315 (serotype M3) chromosome NC_004070 (1900521 bp) | VFDB_setA_NO_Streptococcus.fas | A subset of SetA-vfdb excluding the proteins from *Streptococcus* | Firmicutes | Firmicutes |
| *Vibrio* | Vibrio.fa.p | *V. cholerae O1* biovar El Tor str. N16961 (O1 biovar eltor) chromosome I NC_002505 (2961149 bp) chromosome II NC_002506 (1072315 bp) | VFDB_setA_NO_Vibrio.fas | A subset of SetA-vfdb excluding the proteins from *Vibrio* | Gammaproteobacteria | Gammaproteobacteria |
| *Yersinia* | Yersinia.fa.p | *Y. pestis* CO92 (biovar Orientalis) chromosome NC_003143 (4653728 bp) pMT1 NC_003134 (96210 bp) pCD1 NC_003131 (70305 bp) pPCP1 NC_003132 (9612 bp) | VFDB_setA_NO_Yersinia.fas | A subset of SetA-vfdb excluding the proteins from *Yersinia* | Gammaproteobacteria | Gammaproteobacteria |
